# Arctiin Inhibits Inflammation, Fibrosis, and Tumor Cell Migration in Rats With Ehrlich Solid Carcinoma

**DOI:** 10.7759/cureus.44987

**Published:** 2023-09-10

**Authors:** Bayan M Alfair, Amirah A Jabarti, Shyma S Albalawi, Ahmed E Khodir, Mohammed M Al-Gayyar

**Affiliations:** 1 PharmD Program, University of Tabuk, Tabuk, SAU; 2 Pharmacology and Toxicology, Horus University, New Damietta, EGY; 3 Pharmaceutical Chemistry, Faculty of Pharmacy, University of Tabuk, Tabuk, SAU; 4 Biochemistry, Faculty of Pharmacy, Mansoura University, Mansoura, EGY

**Keywords:** vascular endothelial growth factor (vegf), transforming growth factor (tgf)-β, toll-like receptor 4 (tlr4), signal transducer and activator of transcription 3 (stat3), nlr family pyrin domain containing 3 (nlrp3), ehrlich solid carcinoma (esc), cyclin d1

## Abstract

Background and objectives: ESC or Ehrlich solid carcinoma is a type of tumor originating from a spontaneous mammary adenocarcinoma in mice. It is a highly aggressive and fast-growing carcinoma that can create a solid mass when inserted under the skin. Its solid, undifferentiated form makes it an ideal model for researching cancer biology, tumor immunology, and testing various anti-cancer treatments. Additionally, arctiin has multiple beneficial properties, such as anti-proliferative, anti-oxidative, anti-adipogenic, and anti-bacterial. This study aimed to explore the potential anti-cancer benefits of arctiin in rats with ESC while also analyzing its effects on cell fibrosis markers, tumor cell migration, and inflammasome pathways.

Methods: Rats were given a tumor in their left hind limb via an intramuscular injection consisting of 2×10^6^ cells. After eight days, some of the rats received a daily oral dose of 30 mg/kg of arctiin for three weeks. Muscle samples were observed under an electron microscope or stained with hematoxylin/eosin. Additionally, gene expression and protein levels of toll-like receptor 4 (TLR4), NLR family pyrin domain containing 3 (NLRP3), signal transducer and activator of transcription 3 (STAT3), transforming growth factor (TGF)-β, endothelial growth factor (VEGF), and cyclin D1 were assessed in another part of the muscle samples.

Results: When ESC rats were given arctiin as a treatment, their mean survival time increased and their tumor volume and weight decreased. Additionally, when tumor tissue was examined under an electron microscope, it showed signs of pleomorphic cells, necrosis, nuclear fragmentation, membrane damage with cytoplasmic content spilling, and loss of cellular junction. The stained sections with hematoxylin/eosin showed a dense cellular mass and compressed, degenerated, and atrophied muscle. However, treatment with arctiin improved all these effects. Finally, the expression of TLR4, NLRP3, STAT3, TGF-β, VEGF, and cyclin D1 was significantly reduced with arctiin treatment.

Conclusions: Through the use of arctiin, tumor size and weight were effectively reduced, leading to an increase in the average survival time of rats and an improvement in muscle structure. Additional research has shown that arctiin is able to suppress inflammation, fibrosis, and the migration of tumor cells by inhibiting STAT3, TGF-β1, TLR4, NLRP3, VEGF, and cyclin D1.

## Introduction

Cancer is a serious illness and is the second leading cause of death worldwide. According to a 2018 report by the WHO, it's challenging to treat due to various characteristics related to the disease's pathology and the side effects of chemotherapy, which can include hair loss, fatigue, and nausea, among others [1]. Ehrlich solid carcinoma (ESC) is a tumor that develops from a spontaneous murine mammary adenocarcinoma. It's an aggressive and fast-growing carcinoma that can form a solid mass when inoculated subcutaneously. Because of its undifferentiated solid form, it offers an excellent model for studying cancer biology, tumor immunology, and testing potential anti-cancer therapies [2].

Toll-like receptors (TLRs) are important for protecting the body against infection, but studies conducted recently suggest that TLRs play a crucial role in maintaining tissue homeostasis by regulating the response to injury, including inflammation and tissue repair [3]. TLR4 is a well-studied receptor that can be expressed by cancer cells, allowing them to avoid detection by the immune system and promoting further tumor growth. In fact, TLR4 signaling is also involved in drug resistance and cancer cell proliferation [4]. By blocking or reducing TLR4 activation, developing new treatments that target cancer cells and reverse resistance may be possible.

Lignans are natural compounds that can be found in a variety of seeds, beans, fruits, and vegetables. They are created by pairing phenylpropane structures and are essential to many biological processes, including hormone metabolism, cell differentiation, transformation, and proliferation. One well-known lignan is arctiin, which is found in the *Arctium lappa* L plant. This plant is commonly used as a vegetable in Asian countries and is a popular ingredient in traditional European dishes [5]. Various studies have shown that arctiin possesses several properties, including anti-proliferative, anti-senescence, anti-oxidative, anti-tumor, toxic, anti-adipogenic, and anti-bacterial effects [6]. Therefore, this study aimed to investigate the in vivo anti-cancer effects of arctiin in rats with ESC tumors. Additionally, this study aimed to determine the mechanism of action of arctiin by examining its effects on cell fibrosis markers, tumor cell migration, and inflammasome pathways.

## Materials and methods

Animals’ treatment

This study was carried out on 30 Sprague Dawley rats that weighed between 180 and 200 g. The rats were kept under standard temperature conditions and followed a regular 12-hour light/12-hour dark cycle. Our working protocol with the number P2023-003 was approved by the Research Ethics Committee of Horus University, Faculty of Pharmacy. The rats were divided into three groups, each containing 10 rats. During the experiment period, the control group did not receive any treatment. In contrast, the ESC group was given an intramuscular injection of 0.15 mL 2×10^6^ Ehrlich cells in the thigh of the left hind leg [7-9]. In the ESC group treated with arctiin, we induced ESC in rats. Once a solid tumor appeared on day eight, we administered 30 mg/kg arctiin (St. Louis, MO: Sigma Aldrich Chemicals Co.) by oral gavage and marked it as day zero. The rats were then treated with arctiin for three weeks.

In previous experiments, arctiin was given orally to rats at a dosage of 50 mg/kg to decrease mammary tumors caused by 7,12-dimethylbenz(a)anthracene [10]. Additionally, we conducted initial studies using four different concentrations of arctiin - 30, 50, 70, and 100 mg/kg. The most effective dose was found to be 30 mg/kg, which showed therapeutic benefits even at the lowest dosage.

Sample collection 

A lump on the upper leg of the left hind limb was taken out through surgery. The size and weight of the lump were noted. A piece of muscle tissue was kept in 10% buffered formalin for future studies on morphology and immunohistochemistry. Another part of the tissue was blended with an ice-cold sodium-potassium phosphate buffer and stored at -80°C.

Staining muscle sections with hematoxylin/eosin and immunohistochemistry

In order to study muscle tissues, they were sliced into 5 µm sections and then treated with hematoxylin/eosin stain. To determine the mitotic scores, 10 high-power fields of hematoxylin and eosin-stained samples were analyzed. Additionally, monoclonal anti-TLR4 from MyBioSource, Inc. (San Diego, CA) was used to immune-stain other sections as per our group's previously established methods [11-14]. Our investigations made use of a digital camera-assisted computer system from a Nikon digital camera in Japan.

Specimen preparation for transmission electron microscopy

The samples for electron microscope examination were prepared as described previously by our group [15,16]. Samples measured over 1 mm^3^ were separated and treated with glutaraldehyde at 4°C for 4 hours. Afterward, they were dehydrated using a series of ethanol and propylene oxide and then embedded in epoxy resin. The ultrathin sections were viewed using a JEOL JEM-2100 electron microscope at the Electron Microscope Unit of Mansoura University in Egypt, with observations taken at 160 kV.

Biochemical investigations by enzyme-linked immunosorbent assay (ELISA)

The levels of vascular endothelial growth factor (VEGF), cyclin D1, toll-like receptor 4 (TLR4), NLR family pyrin domain containing 3 (NLRP3), signal transducer and activator of transcription 3 (STAT3), and transforming growth factor (TGF)-β were assessed using ELISA kits from MyBioSource, Inc. (San Diego, CA) in accordance with the manufacturer's guidelines.

RT-PCR investigations

Our team utilized a well-established protocol to examine gene expression in rat muscle. We conducted measurements of specific mRNA levels, such as VEGF, cyclin D1, TLR4, NLRP3, STAT3, and TGF-β using polymerase chain reaction (PCR) primers listed in Table 1 [17-20]. GAPDH was used as our reference control and housekeeping gene.

**Table 1:** Primer sets used to detect gene expression in rats.

Gene symbol	Primer sequence from 5′-3′	Accession number
GAPDH	F: 5′-CCATCAACGACCCCTTCATT-3′ R: 5′-CACGACATACTCAGCACCAGC-3`	NM_017008.4
VEGF	F: 5′-TGCTGCAATGATGAAGCCCT-3′ R: 5′-CTCACAGTGAATGTGGTCACTTA-3′	AY702972.1
Cyclin D1	F: 5′-TCGACGGCCATTACCAATCG-3′ R: 5′-CGCAGACCTCTAGCATCCAG-3′	X75207.1
TLR4	F: 5′-TCAGCTTTGGTCAGTTGGCT-3′ R: 5′-GTCCTTGACCCACTGCAAGA-3′	NM_019178.2
NLRP3	F: 5′-TGCATGCCGTATCTGGTTGT-3′ R: 5′-ACCTCTTGCGAGGGTCTTTG-3′	NM_001191642.1
STAT3	F: 5′-CTGAGGTACAATCCCGCTCG-3′ R: 5′-TCGGTCAGTGTCTTCTGCAC-3′	NM_012747.2
TGF-β	F: 5′-GCTAATGGTGGACCGCAACAAC-3′ R: 5′-CAGCAGCCGGTTACCAAG-3′	NM_021578

Statistical analysis

When we presented quantitative variables, we expressed them as mean±standard error. To compare different groups, we used a one-way analysis of variance (ANOVA) followed by a post hoc Bonferroni correction test. All statistical analyses were conducted using SPSS version 20 (Chicago, IL: IBM Corp.). We considered statistical significance to be p<0.05.

## Results

Chemotherapeutic effects of arctiin

**Figure 1:**
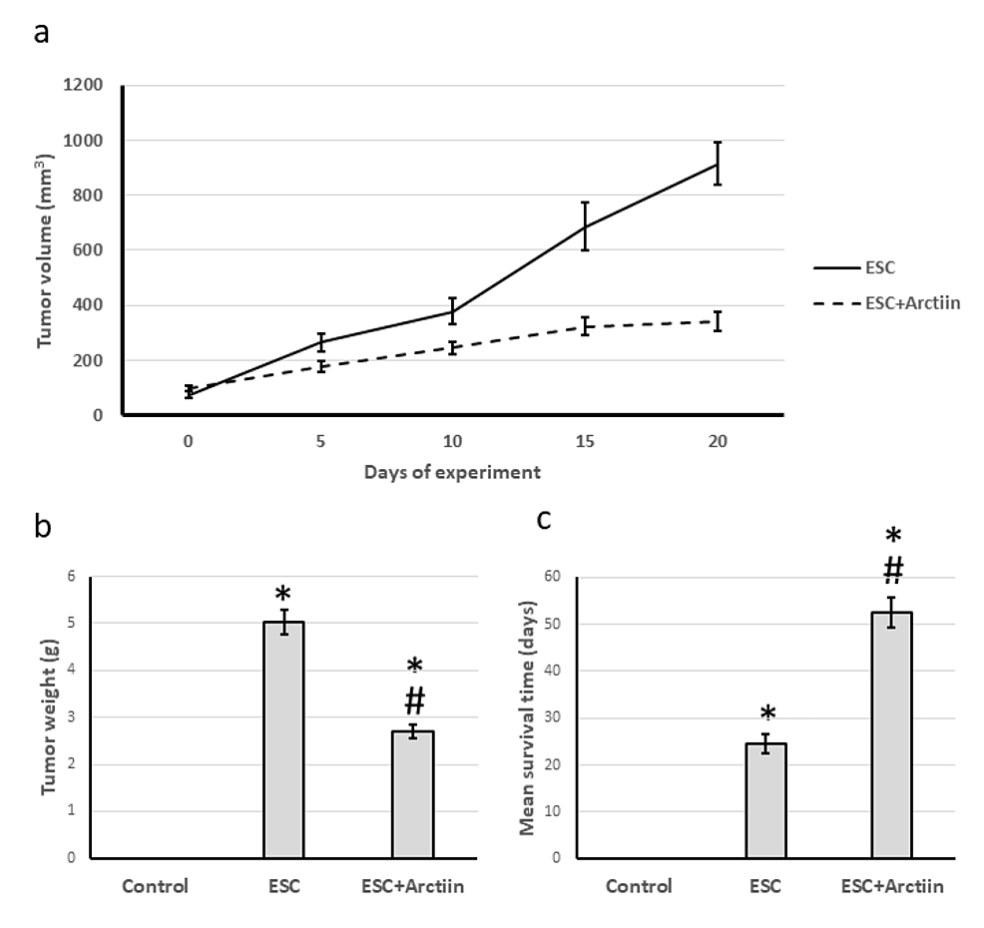
The influence of ESC induced in rats and treatment with 30 mg/kg arctiin on tumor volume (a), tumor weight (b), and mean survival time (c). *Significant difference when compared to the control group at a significance level of p<0.05. #Significant difference when compared to the ESC group at a significance level of p<0.05. ESC: Ehrlich solid carcinoma

First, we examined the direct impact of arctiin on ESC. We found that arctiin had significant protective activity against ESC as it decreased the tumor volume and weight as well as elevated the mean survival time of the rats from 27 days to 53 days (Figures 1a-1c).

Effect of arctiin on muscle cells morphology

**Figure 2:**
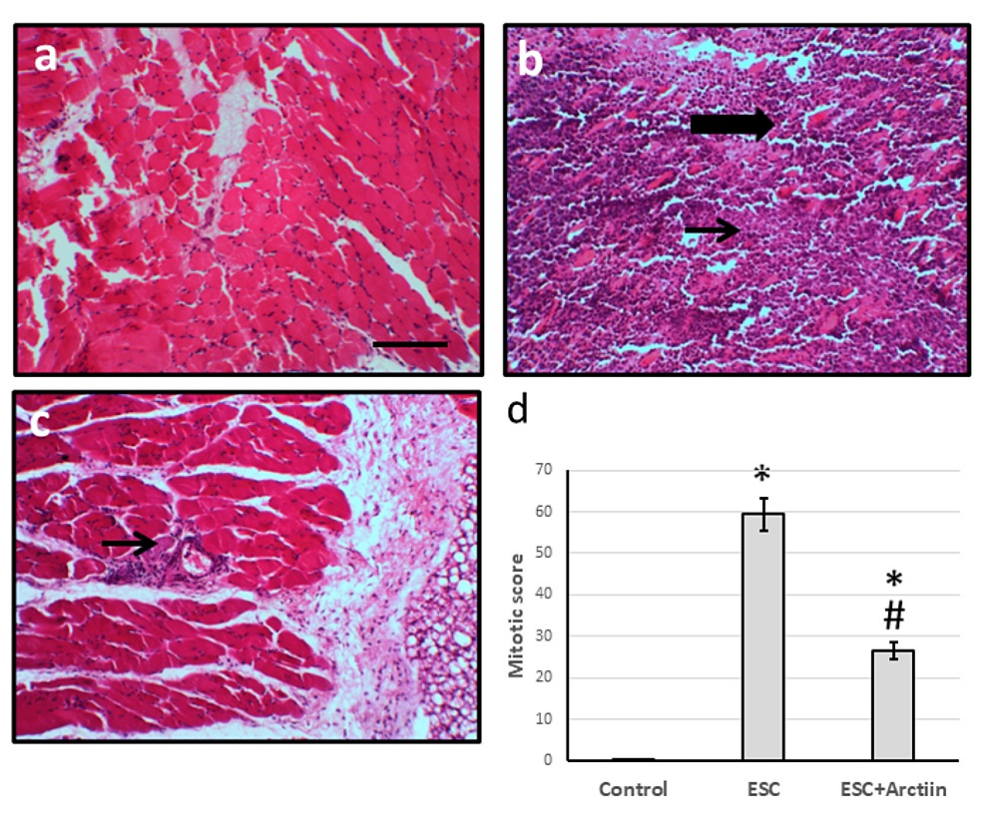
Muscle sections stained with hematoxylin and eosin in the control group (a), ESC (b), and ESC treated with 30 mg/kg arctiin (c). The mitotic figure was determined in 10 fields of high field power and expressed as mean ± standard deviation (d). Thin arrows represented densely cellular mass and thick arrows represented compressed, degenerated, and atrophied muscle. Scale bar 100 μm. *Significant difference when compared with the control group at p<0.05. #Significant difference when compared with ESC group at p<0.05. ESC: Ehrlich solid carcinoma

The control group's micro images, stained with hematoxylin/eosin, displayed a normal appearance of muscle tissue. In contrast, the micro images of ESC showed a dense cellular mass (thin arrows) and compressed, degenerated, and atrophied muscle (thick arrows). However, after treatment with arctiin, the muscle cells' structure greatly improved. Additionally, compared to the ESC group, arctiin significantly decreased the fibrotic score (Figures 2a-2d). These results suggest that arctiin may have an anti-proliferative effect on cancer cells.

**Figure 3:**
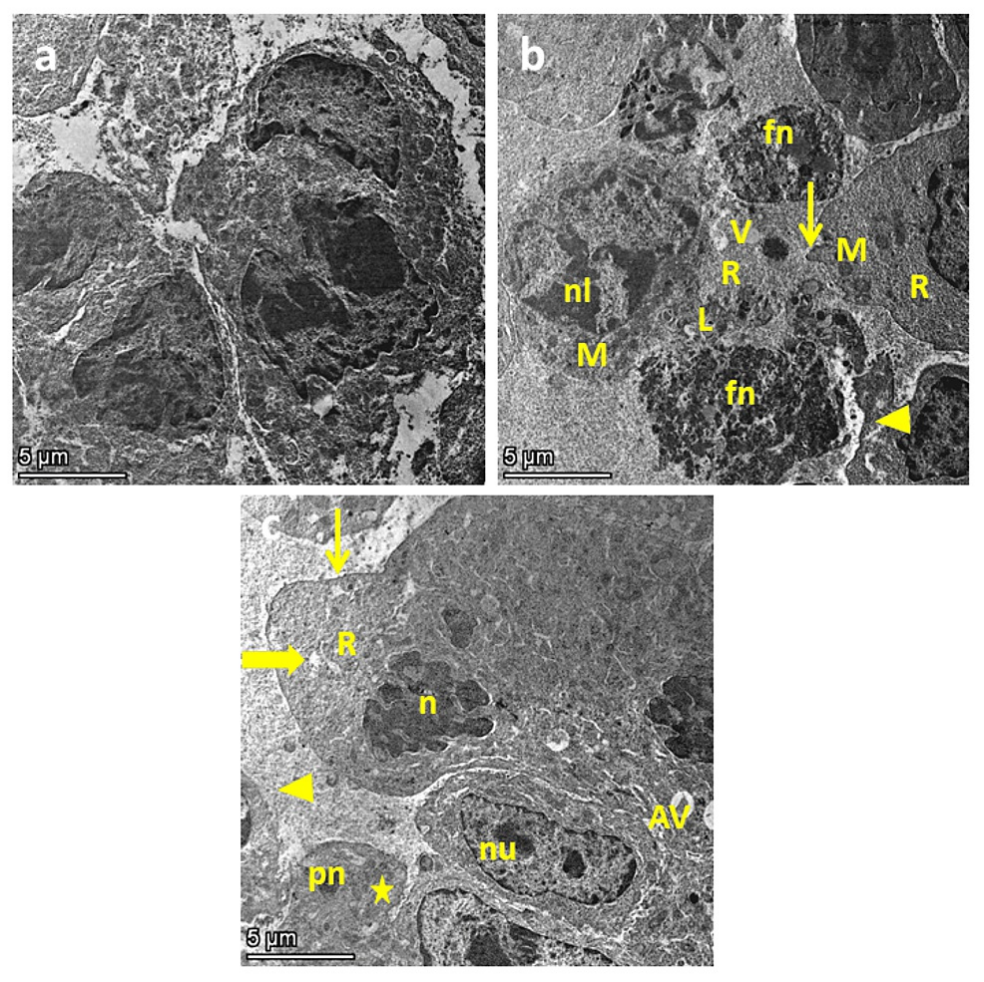
Muscle sections observed under an electron microscope in the control group (a), ESC (b), and ESC treated with 30 mg/kg arctiin (c). Thick arrows represent enlarged pleomorphic cells, thin arrows represent irregular plasma membrane, letter n represents irregular nuclei, letter R represents extensive cytoplasmic ribosomes, arrowhead represents widened intercellular space, star represents necrotic cells, pn represents pyknosis and membrane rupture, nu represents irregular nucleus with two prominent nucleoli. The scale bar is 5 μm. ESC: Ehrlich solid carcinoma; AV: autophagic vacuoles

In transmission electron microscopy images of muscle samples from the ESC group, there were cells displaying a variety of characteristics. It showed pleomorphic cells, two cells are necrotic with nuclear fragmentation (fn), abundant electron-dense secondary lysosomes (L) and ribosomes (R), membrane damage with spilling of cytoplasmic content, and loss of cellular junction (arrowhead). Other cells show nuclear lobulation (nl) with cytoplasmic mitochondrial irregularity (M) and membrane blebs (thin arrow). While investigating samples from ESC, rats treated with arctiin showed improvement in the cell structure (Figures 3a-3c). 

Effect of arctiin on inflammasomes

**Figure 4:**
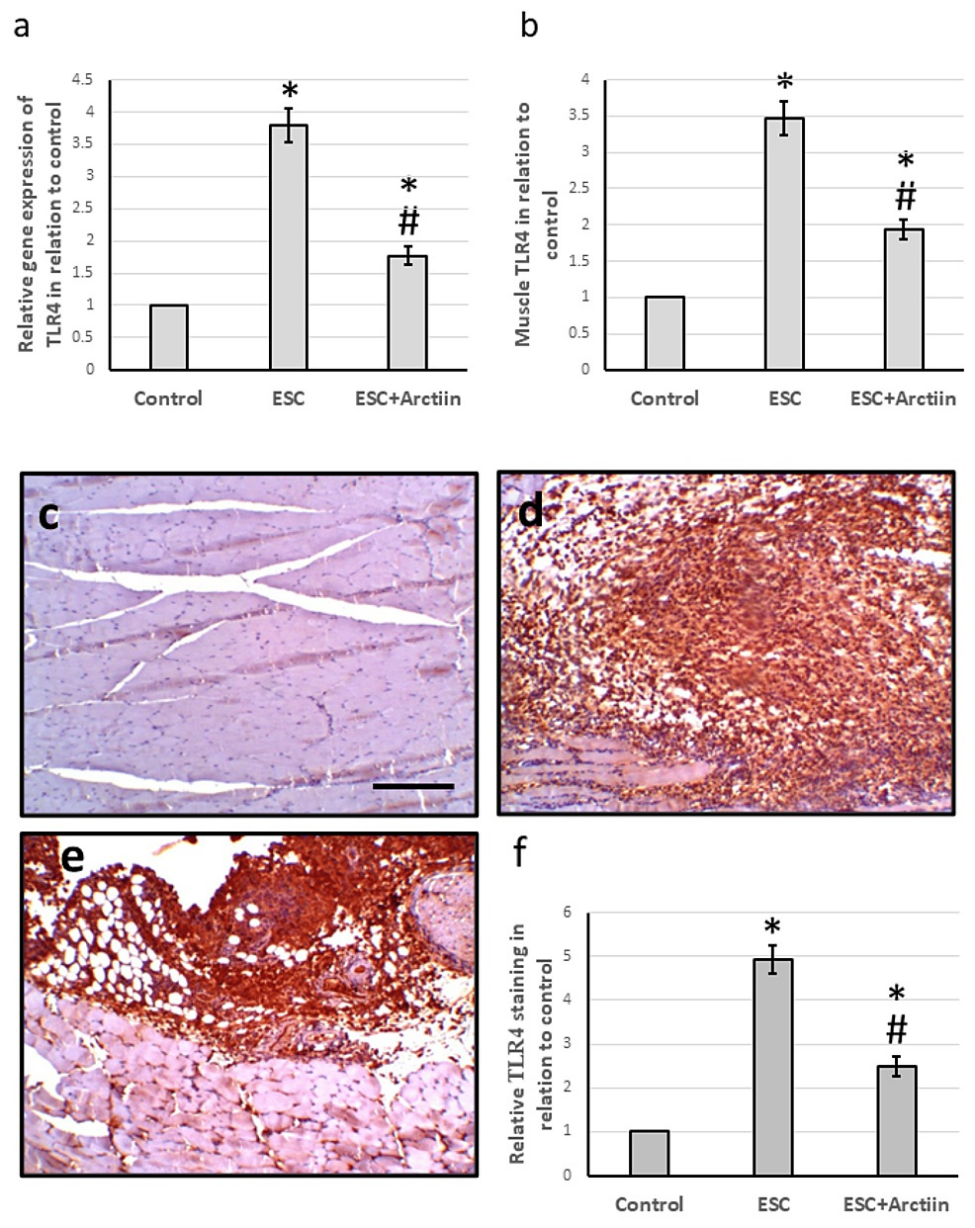
The influence of ESC induced in rats and treatment with 30 mg/kg arctiin on gene expression of TLR4 (a) and its muscle protein level (b). Muscle sections stained with TLR4 antibodies in the control group (c), ESC group (d), and ESC group treated with arctiin (e). Immunohistochemistry score of positive staining (f). Scale bar 100 μm. *Significant difference when compared to the control group at a significance level of p<0.05. #Significant difference when compared to the ESC group at a significance level of p<0.05. ESC: Ehrlich solid carcinoma; TLR4: toll-like receptor 4

The rats that were given ESC had higher levels of TLR4 gene expression (3.79 times) and protein levels (3.47 times) in their muscles than the control group. TLR4 immune staining also showed higher levels of TLR4 expression in the muscles of ESC rats. However, treatment with arctiin significantly reduced both gene expression and protein levels, as well as TLR4 immunostaining, in ESC rats. These findings are shown in Figures 4a-4f.

**Figure 5:**
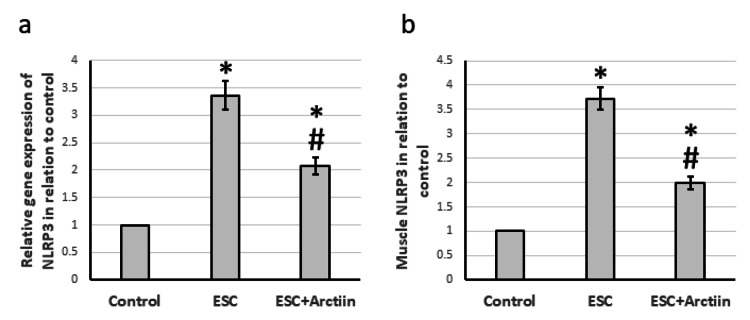
The influence of ESC induced in rats and treatment with 30 mg/kg arctiin on gene expression of NLRP3 (a) as well as the protein levels of NLRP3 (b). *Significant difference when compared to the control group at a significance level of p<0.05. #Significant difference when compared to the ESC group at a significance level of p<0.05. ESC: Ehrlich solid carcinoma; NLRP3: NLR family pyrin domain containing 3

Additionally, rats with ESC had significantly higher levels of NLRP3 gene expression (3.36-fold) and protein levels (3.72-fold) in their muscles compared to the control group. Treatment of ESC rats with arctiin significantly decreased both gene expression and protein levels of NLRP3 compared to ESC rats (Figures 5a, 5b).

Effect of arctiin on cells fibrosis markers

**Figure 6:**
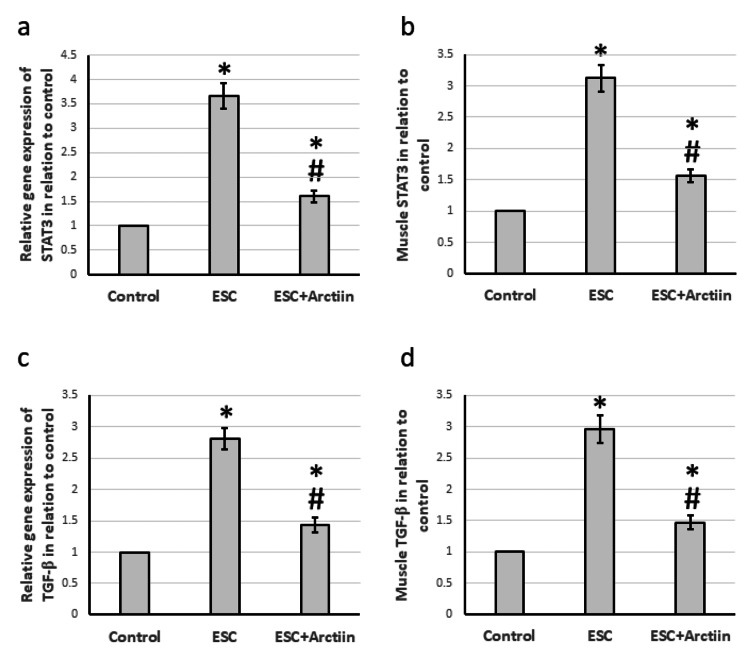
The influence of ESC induced in rats and treatment with 30 mg/kg arctiin on gene expression of STAT3 (a), and TGF-β (c) as well as the protein levels of STAT3 (b) and TGF-β (d). *Significant difference when compared to the control group at a significance level of p<0.05. #Significant difference when compared to the ESC group at a significance level of p<0.05. ESC: Ehrlich solid carcinoma; STAT3: signal transducer and activator of transcription 3; TGF-β: transforming growth factor

When ESC rats were studied, it was found that ESC resulted in a 3.67- and 2.81-fold increase in the gene expression of STAT3 and TGF-β1. This was associated with a 3.12- and 2.96-fold increase in the protein levels of both compounds, respectively, as compared with the control group. However, when ESC rats were treated with arctiin, both gene expression and protein levels of STAT3 and TGF-β1 were significantly reduced as compared with ESC rats (Figures 6a-6d).

Effect of arctiin on cells’ migration markers

**Figure 7:**
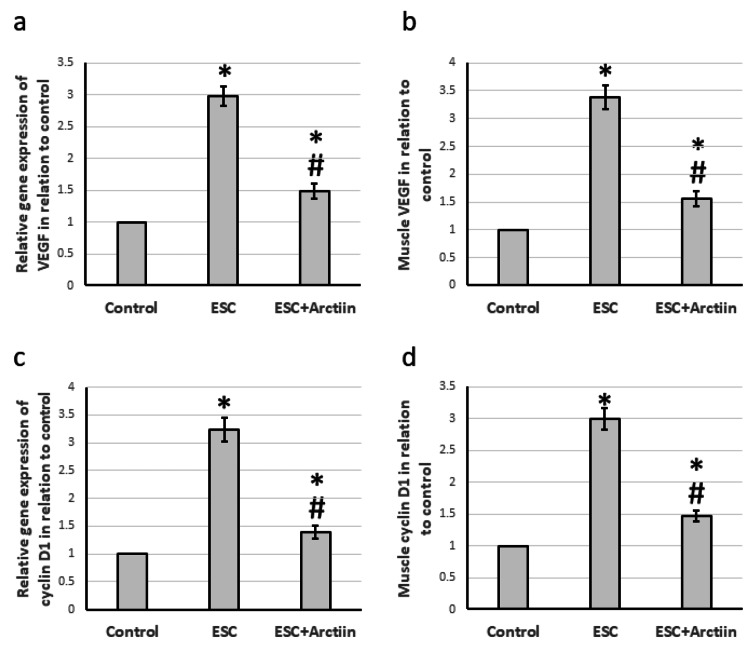
The influence of ESC induced in rats and treatment with 30 mg/kg arctiin on gene expression of VEGF (a), and cyclin D1 (c) as well as the protein levels of VEGF (b) and cyclin D1 (d). *Significant difference when compared to the control group at a significance level of p<0.05. #Significant difference when compared to the ESC group at a significance level of p<0.05. ESC: Ehrlich solid carcinoma; VEGF: vascular endothelial growth factor

Arctiin has a protective effect on ESC and regulates various signaling pathways. It can regulate markers responsible for angiogenesis and migration of tumor cells. Post ESC, there was a significant increase in the gene expression of VEGF and cyclin D1, by 2.98- to 3.23-fold, respectively. This led to a 3.38- and 2.99-fold increase in the levels of VEGF and cyclin D1 in the muscle compared to the control group. However, in rats treated with arctiin, these effects were reversed (Figures 7a-7d).

## Discussion

Cancer is a major concern worldwide, ranking as the second leading cause of death in the United States. In 2020, there were over 18 million cases of cancer diagnosed globally. Denmark had the highest incidence rate of cancer in both males and females, with 334.9 people per 100,000 affected. Meanwhile, Mongolia reported the highest cancer-related mortality rate, with 175.9 people per 100,000 [21]. Cancer treatment can cause severe physical and emotional symptoms, leading to a decrease in the quality of life and depression. Chemotherapy, in particular, can be expensive and result in serious side effects. Our research aimed to explore the potential of arctiin as a chemotherapeutic agent. We used the ESC tumor model, which is a rapid-growth in vivo experimental model for testing anti-tumor compounds. After implanting Ehrlich cancer cells in rats, we observed tumor growth over the course of three weeks. The diagnosis was confirmed by weighing the tumor after separation from the leg and inspection of micro-images stained with hematoxylin/eosin, showing a densely cellular mass and compressed, degenerated, and atrophied muscle. In addition, investigation of ESC samples under an electron microscope showed pleomorphic cells, necrosis, nuclear fragmentation, membrane damage with spilling of cytoplasmic content, and loss of cellular junction. However, treating the ESC rats with arctiin significantly reduced the tumor volume and weight while prolonging the rats' mean survival time from 27 to 53 days due to the ability of arctiin to reduce tumor growth and inhibition of tissue damage as a result of fibrosis and tumor migration. Furthermore, arctiin restored the normal structure of muscle cells, as seen in muscle sections stained with hematoxylin/eosin. Previous studies have shown arctiin to have chemotherapeutic effects against cervical cancer by inhibiting the PI3K/AKT pathway; however, this study is the first to illustrate arctiin's anticancer effects on ESC [22].

Our research examined the chemotherapeutic activity of arctiin and focused on the TLR4 pathway, which is known to play a crucial role in tumor activation and carcinogenesis. TLR4 acts as a mediator between innate and adaptive immunity and is expressed in immune cells, epithelial/endothelial cells, and tumor cells [23]. The NLRP3 pathway is also associated with TLR4 and leads to the activation of pro-inflammatory cytokines, which are highly involved in tumorigenesis. Previous studies have shown that both TLR4 and NLRP3 are upregulated in many human cancers [24]. However, our research showed that the expression of both TLR4 and NLRP3 was significantly reduced in ESC when rats were treated with arctiin. Arctiin was found to inhibit TLR4 and NLRP3 in silica-induced oxidative injury of lung by inhibiting macrophage M0 polarization to M1, alleviating proinflammatory response, and reducing the release of inflammatory factors. This blocking of macrophage polarization may be associated with the blocking of TLR-4/NLRP3 signaling [25]. While previous studies have illustrated the ability of arctiin to inhibit TLR4/NLRP3 in other cases, our study is the first to demonstrate this ability in ESC.

Fibrosis is the end result of many chronic inflammatory diseases, and a molecule called TGF-β is responsible for regulating it. TGF-β serves as a cytokine and manages collagen formation and the deposition of collagen proteins in cardiac fibroblasts. It also controls the deposition of extracellular matrix protein [26]. Another important factor in fibrosis is STAT3, which is involved in central nervous system development, immune response, stem cell maintenance, and tumorigenesis. STAT3 helps with the proliferation and maintenance of certain tumors but inhibiting it can lead to cell cycle arrest and apoptosis [27]. Our study found an increase in the expression of both TGF-β1 and STAT3 in the ESC group. However, treating ESC rats with arctiin significantly reduced the expression of both TGF-β1 and STAT3. This deactivation of STAT-3 can suppress cancer-related inflammation and reduce the immune-suppressive environment of tumors, leading to the activation of antitumor immunity and the enhancement of cytototoxic T-cell effector functions. Arctiin has been reported to inhibit STAT3 phosphorylation at the tyrosine 705 residue and has shown potential in treating human multiple myeloma cells [28]. Its use in our study may provide a potential therapeutic benefit in reducing fibrosis and inflammation. Multiple studies have demonstrated that STAT3 plays a crucial role in regulating various target genes during tumor development and progression, including VEGF, cyclin D1, and Bcl-xL.

Tumor growth and metastasis are dependent on neoangiogenesis, which is facilitated by VEGF. Oncologists have been exploring ways to induce tumor cell death by cutting off the blood supply. VEGF also contributes to immunosuppression, which is linked to the Treg population and inversely related to the CD8+ cytotoxic T cell population [29]. Cyclin D1, a regulator of cell proliferation, is frequently overexpressed in tumors. In colorectal cancer cells, STAT3 can directly target the promoter of cyclin D1. In ESC rats, we observed high levels of both VEGF and cyclin D1 expression, which were reduced by administering arctiin. Previous research has shown that arctiin can inhibit VEGF expression in human retinal capillary endothelial cells [30] and cyclin D1 expression in MC3T3-E1 cells that have undergone osteogenic differentiation [31]. Nevertheless, this study is the first to demonstrate arctiin's ability to inhibit the expression of VEGF and cyclin D1 in ESC.

**Figure 8:**
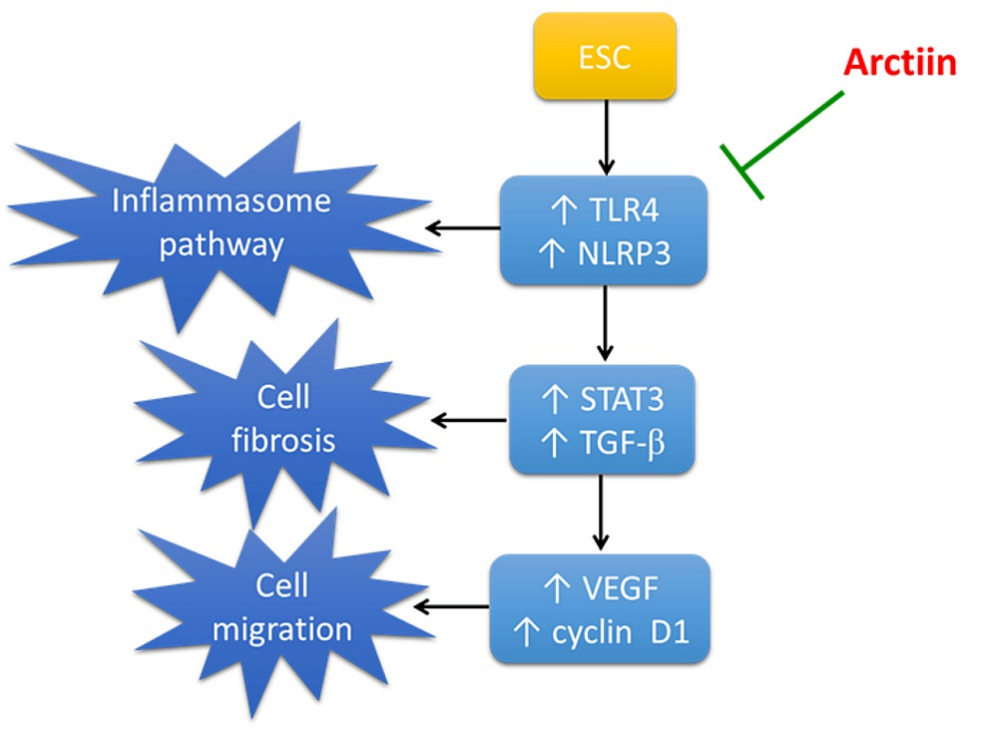
Schematic presentation of the mechanism of action of arctiin in ESC. ESC: Ehrlich solid carcinoma; NLRP3: NLR family pyrin domain containing 3; STAT3: signal transducer and activator of transcription 3; TLR4: toll-like receptor 4; TGF-β: transforming growth factor-β; VEGF: vascular endothelial growth factor The image is created by the authors of this study.

Arctiin is a promising chemotherapy alternative due to its natural origin, affordability, and safety. Figure 8 outlines how it therapeutically affects ESC. However, current research has some limitations. For instance, rats have different metabolic processes than humans, which can lead to varying drug effects. Additionally, there are numerous cancer induction animal models, but our study only utilized one method.

## Conclusions

Our research aimed to investigate whether arctiin could be used as a chemotherapy agent for ESC. Our findings indicate that arctiin was effective in reducing tumor size and weight, as well as increasing the average survival time of rats. We also observed an improvement in muscle tissue structure when examined under an electron microscope or stained with hematoxylin/eosin. Further investigation demonstrated that arctiin suppresses inflammation, fibrosis, and tumor cell migration by inhibiting STAT3, TGF-β1, TLR4, NLRP3, VEGF, and cyclin D1. Based on these results, we conclude that arctiin shows potential as a chemotherapy treatment for ESC, at least in the Murine model.
